# Feasibility of dried blood spot for hepatitis C diagnosis in vulnerable subjects and people living in remote areas from Brazil

**DOI:** 10.1186/s12879-022-07717-4

**Published:** 2022-10-27

**Authors:** Livia Melo Villar, Marjorie Parra de Lima, Helena Medina Cruz, Vanessa Salete de Paula, Leticia de Paula Scalioni, Geane Lopes Flores, Filipe Anibal Carvalho-Costa, Cynara Carvalho Parente, Maria Rosangela Cunha Duarte Coelho, Ana Cecilia Cavalcanti de Albuquerque, Flavio Augusto Pádua Milagres, Marcelo Santos Cruz, Tarcisio Matos Andrade, Ana Rita Coimbra Motta-Castro, Jurema Corrêa da Mota, Lia Laura Lewis-Ximenez, Francisco Inácio Bastos

**Affiliations:** 1 Laboratory of Viral Hepatitis, Oswaldo Cruz Institute, FIOCRUZ, Helio and Peggy Pereira Pavillion, Ground Floor, Room B09, v. Brasil, 4365, Manguinhos, Rio de Janeiro, RJ 210360-040 Brazil; 2 Estácio de Sá University, Resende, Rio de Janeiro, Brazil; 3 Molecular Virology Laboratory, Oswaldo Cruz Institute, FIOCRUZ, Rio de Janeiro, Brazil; 4 Laboratory of Epidemiology and Molecular Systematics, Oswaldo Cruz Institute, FIOCRUZ, Rio de Janeiro, Brazil; 5 Medicine Faculty, Universidade Federal do Ceará, Sobral, Ceará Brazil; 6 Virology Sector, Laboratory of Immunopathology Keizo Asami, Federal University of Pernambuco, Recife, Pernambuco Brazil; 7 Centro Universitário Tabosa de Almeida, Caruaru, Pernambuco, Brazil; 8 Medicine Faculty, Federal University of Tocantins, Palmas, Brazil; 9 Institute of Psychiatry, Federal University of Rio de Janeiro, Rio de Janeiro, Brazil; 10 Department of Community and Family Health, Federal University of Bahia, SalvadorBahia, 40110-100 Brazil; 11 Federal University of Mato Grosso do Sul and FIOCRUZ-MS, Campo Grande, MS Brazil; 12 Institute of Communication and Scientific Information and Technology for Health, Oswaldo Cruz Foundation, Rio de Janeiro, Brazil

**Keywords:** Hepatitis C, Dried blood spot, Diagnosis, Vulnerable populations

## Abstract

**Background:**

Agile, accessible and cheap diagnosis of hepatitis C virus (HCV) infection is essential to achieve the elimination of this infection, worldwide, as mandated by the World Health Organzation as part of its strategy for 2030. Dried blood spots (DBS) can be an attractive alternative for sample collection among people living in remote areas and vulnerable populations due to the less invasive collection, its biosafety, and storage & transportation of samples at room temperature.

**Design:**

This study aims to estimate the usefulness of dried blood spot samples for the diagnosis and the assessment of HCV infection rates in three different settings in Brazil. Cross-sectional analysis of a sample collection from different populations, aiming to assess the performance of the testing algorithms and respective procedures among different populations with diverse background infection rates.

**Methods:**

We reported the evaluation of DBS as alternative samples for detecting anti-HCV in different groups in real life conditions: (I) Vulnerable subjects living in remote areas of Southeast, North and Northeast Brazil (n = 1464); (II) Beauticians (n = 288); (III) People who use non-injectable drugs (n = 201); (IV) patients referred to outpatient care (n = 275).

**Results:**

General assay accuracy was 99%, with a weighted kappa value of 0.9, showing an excellent performance. Sensitivities ranged from 87.5% to 100.0% between groups and specificities were above 99.2%. A total of 194 individuals had HCV RNA in serum and concordance of anti-HCV detection in DBS was 98.4%.

**Conclusions:**

DBS samples could be used for anti-HCV detection in different populations recruited in real life conditions and ambulatory settings, with a high overall sensitivity and specificity.

**Supplementary Information:**

The online version contains supplementary material available at 10.1186/s12879-022-07717-4.

## Background

Hepatitis C virus (HCV) is responsible for 58 million of chronic cases all over the world [1]. Of those with chronic HCV infection, the risk of cirrhosis ranges between 15 and 30% over 20 years [1]. HCV prevalence may vary according geographical region, risk behaviors, and the interaction of individuals and groups with social networks where different habits are more or less frequent, background infection rates differ and genetic clusters may or may not exist [2–4].

The World Health Organization has proposed the elimination of chronic viral hepatitis C as a key public health goal by 2030 [5]. Recent papers have discussed whether this is a feasible goal and have focused instead on micro-elimination [6]. To achieve both the original goal of global elimination or micro-elimination, it is important to diagnose and to counsel people at risk, with prompt referral to treatment and the systematic use of different regimens of direct antivirals [7].

Diagnosis of HCV infection is quite difficult in low resource areas due to the absence of infrastructure or specialized personnel for blood collection and testing. Dried blood spot samples (DBS) could be an alternative specimen to serum for hepatitis testing that might increase the access of diagnosis in remote areas, save dollars from contrained budgets and save hours and effort of scarce human resources [8].

Some studies have demonstrated HCV detection using DBS, but most of them used few samples from high endemicity areas or clinical settings. Findings on sensitivity and specificity have varied from 92.6–100.0% to 98–100%, respectively [9–19].

HCV prevalence studies are crucial to elaborate control and prevention measures. Most of HCV prevalence studies have been conducted in high-risk groups, such as, people living with human immunodeficiency virus (HIV), subjects under haemodialysis, people who use drugs (PWUD) [20, 21]. In Brazil, some regions such as Amazonia and Pantanal Wetlands have been exposed to fires that deeply affect the environment and the health of population [22], a dire situation that became much worse in recent years [23]. In these areas live several indigenous people (the vast majority of the more than 300 different indigenous populations/ethnicities [available at: https://pib.socioambiental.org/pt/Quadro_Geral_dos_Povos]), quilombo reminiscents (“quilombolas” [roughly translated as “marooners”]; [24] and riverine communities, but there are scarce data about HCV prevalence and HCV transmission dynamics. Both are pivotal to inform sound policies to curb HCV spread.

Among the populations usually targeted by the micro elimination, we should highlight people under haemodialysis and people who use some illicit drugs, especially when they self-administer such substances using different drug equipment (e.g. needles and syringes, as well as devices used to smoke or snort powder cocaine and crack cocaine) [25, 26]. PWUD are difficult to reach for HCV testing due to poor conditions and high mobility. HCV prevalences varies from 2.8 to 12.6% among subjects under hemodialysis [20, 27, 28] and from 1.3% to 4.5% among people who use crack cocaine in Brazil [21, 29]. DBS testing for anti-HCV could be an important tool to increase the access to diagnosis in these individuals what could be important to micro elimination programs of HCV infection.

This study aims to estimated the usefulness of dried blood spot samples for the diagnosis and the assessment of HCV infection rates in three different settings in Brazil. We hyphotesized that a comprehensive assessment of test performance in context, with different populations may represent a helpful proof of concept and evidence for clinicians and policymakers.

## Materials and methods

### Study design

The present study performed a cross-sectional analysis of a panel of biological samples that are part of a set of studies that analyzed vulnerable populations and people living in remote areas of Brazil, at risk of HCV infection. FIOCRUZ panel of biological samples is a years-long initiative, planed in advance to provide reserachers with the most comprehensive set of biological samples from different populations, with an emphasis on vulnerable and hard-to-reach segments with moderate to high background infection rates. The overall purpose is to simulate as far as possible real life conditions and to avert by all means the recourse to ad hoc alternatives.

### Study population

The study comprises the analysis of a panel of biological samples as diverse as possible, aiming to emulate the diversity of real life conditions affecting vulnerable populations in context. They are composed by pools of convenience samples and following is a detailed description these populations.

Group I: 1042 Vulnerable subjects living in remote areas composed by 329 subjects living at Pantanal region of Mato Grosso do Sul State located at Mid West region of Brazil; 582 indigenous people from Tocantins State and 131 individuals from Southeast region (the municipality of Nova Iguaçu, located in the outskirts of Rio de Janeiro Metropolitan Areas). All individuals were living in dire poverty, lack even comprehensive health care. and did not have viral hepatitis diagnosed or symptoms at the moment of inclusion in the study.

Group II: A total of 288 beauty professionals from Southeast Brazil (Rio de Janeiro State). Beauty professionals were manicures, pedicure, beauty dressers and epilators.

Group III: 201 non-injectable drug users (NIDU) from Southeast and Northeast region of Brazil. NIDU are defined as those people who use substances, but did not report injectable drug use in the last 12 months prior to the inclusion in the study and should not be under the influence of drugs at the moment of recruitment. Many of them are ex-injectors, nowadays a very tiny population in Brazil, as explained in detail elsewhere [30].

The groups II and III aged 18y old or more (i.e. comprising with Brazilian legal age and then with full informed consent).

Group IV: A total of 275 patients referred to Viral Hepatitis Ambulatory at the Oswaldo Cruz Institute in Rio de Janeiro. All patients were ≥ 18 year old and are confirmed cases of viral hepatitis or HCV infection, using serological and serological molecular tests. Exclusion criterion comprised the impossibility to provide serum samples.

Additionally, a total of 422 individuals from North and Northeast States (Amazonas, Pernambuco, Ceará and Piaui) gave only DBS samples and anti-HCV detection was presented in Additional file 3: Appendix.

All individuals gave informed consent to participate in the study. This study was approved by the Ethics Committee of FIOCRUZ under the number #889.582 and followed according to the ethical guidelines of the 1975 Declaration of Helsinki.

### Sample collection

Blood samples were collected by venipuncture to obtain whole blood and serum samples from all individuals, exception individuals from Northeast region (Group 1) that only gave DBS samples obtained by digital punch. DBS samples were obtained by spotting 3–5 drops (approximately 75 μL) of whole blood onto Whatman filter paper (Whatman Protein SaverTM n° 903, G&E) to fill completely 12-mm preprinted circular paper disks and processed as described previously [14, 31].

### Sampling procedures

Despite the challenges imposed on statistical inference secondary to the analysis of non-probability samples (reference by Elliott and Valliant to be numbered), there is here a clear trade-off.

The authors deliberately chose to err on the “extreme” of comprehensive statistical inference, but to assess hard-to-reach, sparse and marginalized populations. There is no way to obtain a comprehensive list of such populations.

The classical probability assumptions such as the precise definition of selection probability for all participants, the exclusion of the possibility of having a potential participant with a zero probability of being selected, as well as full equiprobable selection of all putative participants cannot be fulfilled (please, see a basic definition of such assumptions at: https://www.statisticshowto.com/classical-probability-definition/).

But the costs to be incurred to obtain an elusive probability-sample or an optimal consecutive series of scattered inidividuals would be translated into the exclusion of all populations under study.

For this very reason, statistical inference has been used for the sole purposes of diagnostic accuracy studies, with no attempt to make naïve generalizations or to present findings with a precision that may ignore biases secondary to sampling errors. Our analysis are compatible with the limitations of convenience samples, as has been observed for all studies targeting hard-to-reach, sparse populations, worldwide.

### Laboratory tests

Serum and DBS samples were submitted to anti-HCV-EIA (Murex HCVAb, Diasorin, Italy) where supplier’s information was followed for serum samples. For DBS samples, 100 µl of eluate and 100 µl of sample diluent were used. ROC curve analysis was used to determine a cut off value of EIA assay (index test) in DBS to distinguish reactive from non-reactive samples. Cut off value of EIA assay in serum samples was calculated using the formule: CNX + 0.500 where CNX is the mean of DOs of negative serum controls. Clinical information and reference standard results were not available to the performers of the index test to make the results more reliable. Reactive or indeterminate samples were retested in duplicate and samples with discordant results were excluded from the study.

Serum anti-HCV reactive samples were tested for the presence of HCV RNA using Abbott Real time HCV Assay (Abbott, Des Plaines, Illinois, USA) along to Abbott Sample Preparation System reagents and Abbott m2000sp and m2000rt instruments. The gold standard for anti-HCV detection in DBS was anti-HCV testing in serum samples. ELISA was the reference test for this study and choosed since it is the method employed all over the world to detect antibodies in laboratory settings.

### Data analysis

Clinical and epidemiological data were coded and entered into a datasheet (Excel 2010, Microsoft Inc., USA). The detection of anti-HCV antibodies in the serum samples by the EIAs was used as the gold standard for the assessment of the sensitivity, the specificity, the positive predictive value (PPV), and the negative predictive value (NPV), accuracy of the assay in DBS, and respective 95% confidence intervals (95%CIs) were calculated. The weighted Kappa coefficient (wK) was used to assess the degree of agreement between serum and DBS results, as mandated for non-ordinal variables (https://www.ibm.com/docs/en/spss-statistics/SaaS?topic=features-weighted-kappa).

ROC (Receiver Operating Curves) wer fitted, an their integrals calculated, which are geometrically equivalent to the Area Under the Curve (AUC).

MedCalc^®^ and GraphPad instat^®^ were used for these calculations, with additional analyses performed in R (R Core Team (2021). R: A language and environment for statistical computing. R Foundation for Statistical Computing, Vienna, Austria. URL https://www.R-project.org/).

## Results

### Demographic and behavior factors for HCV in the population studied

A total of 1,806 individuals were recruited for this study. Main characteristics of the population is presented in Table 1. Overall, there was a predominance of females (53.4%), individuals had got up to 14 years of education (25.9%), with an average monthly income of U$202.10–606.31 (31.3% of the sample).

**Table 1:** Socio-demographic according to each group in the study

Variable	Total population	Group I	Group II	Group III	Group IV
	(n = 1,806)	(n = 1,042)	(n = 288)	(n = 201)	(n = 275)
	n (%)	n (%)	n (%)	n (%)	n (%)
Gender*					
Female	964 (53.4)	552 (53.0)	213 (74.0)	36 (17.9)	163 (59.3)
Male	799 (44.2)	449 (43.1)	74 (25.7)	165 (82.1)	111 (40.4)
Age (years)	33.4 ± 18.9	28.7 ± 18.9	37.2 ± 14.5	21.6 ± 3.8	54.3 ± 10.6
Years of education*					
None	21 (1.2)	11 (1.1)	–	5 (2.5)	5 (1.8)
Up to 2 years	115 (6.4)	16 (1.5)	24 (8.3)	17 (8.5)	58 (21.1)
Up to 11 years	382 (21.2)	160 (15.4)	36 (12.5)	119 (59.2)	67 (24.4)
Up to 14 years	468 (25.9)	148 (14.2)	176 (61.1)	39 (19.4)	105 (38.2)
More than 14 years	159 (8.8)	68 (6.5)	52 (18.1)	7 (3.5)	32 (11.6)
Monthly family income*					
Low (< US$202.10)	71 (3.9)	48 (4.6)	9 (3.1)	4 (2.0)	10 (3.6)
Intermediate (U$202.10–606.31)	566 (31.3)	173 (16.6)	158 (54.9)	60 (29.9)	175 (63.6)
High(> U$606.31)	338 (18.7)	130 (12.5)	117 (40.6)	19 (9.5)	72 (26.2)

FIOCRUZ Viral Hepatitis Laboratory, Biological Samples Panel, 2020

### Performance of anti-HCV in DBS samples according study groups

**Fig. 1:**
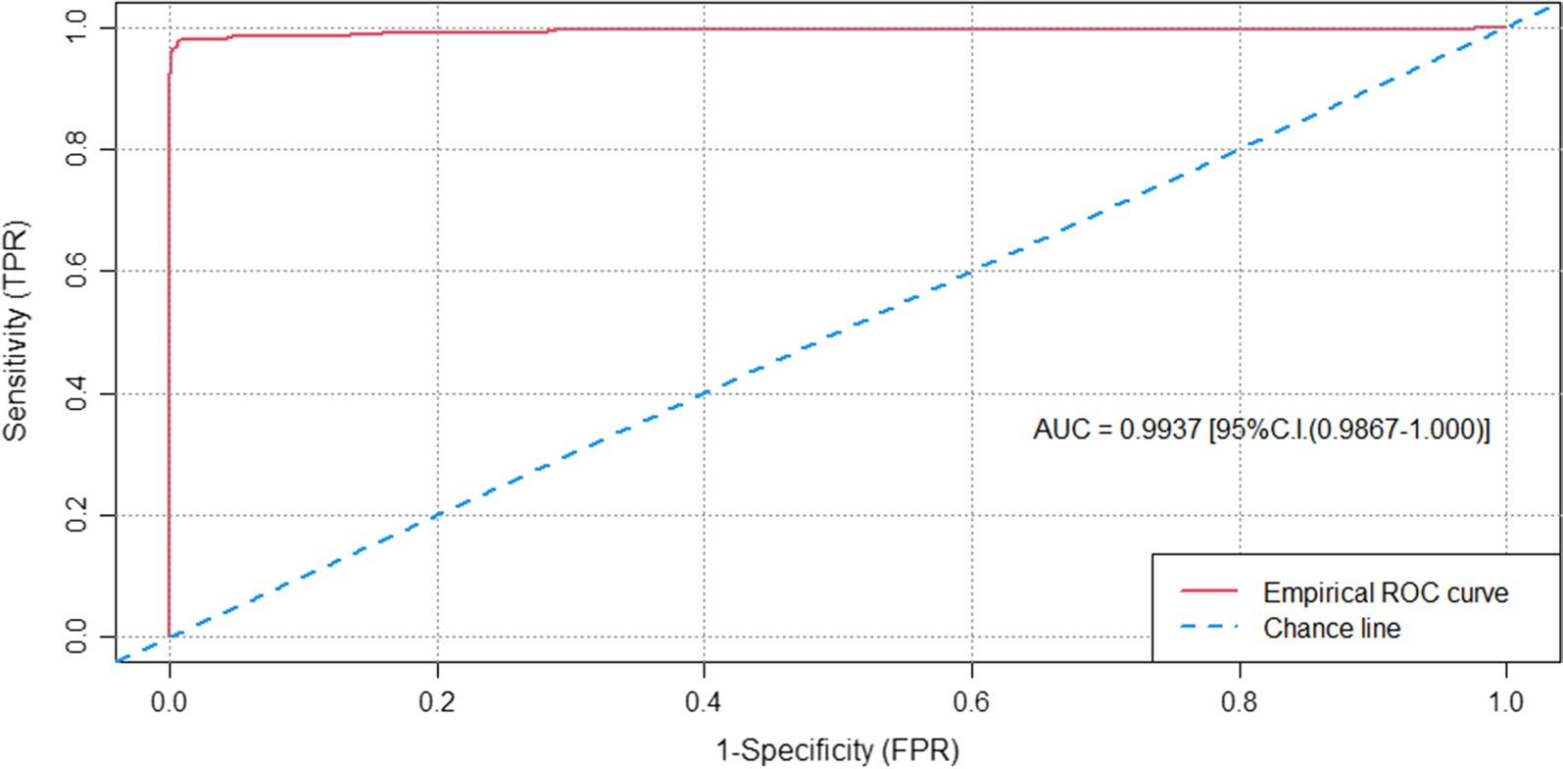
Receiver operating characteristic (ROC) curve of the Performance of anti-HCV in DBS samples

To determine a cut-off value, OD values of DBS samples were compared to results of serum samples to make ROC curve analysis and a value of 0.11 was estimated to distinguish positive and negative samples. Reactive samples should present OD values above 0.11 (Fig. 1).

Overall, 255 anti-HCV reactive serum samples were detected and 250 of them were also reactive in DBS, yielding a sensitivity of 98.0%. On the other hand, anti-HCV was not detected in 1551 serum samples and in 1539 paired DBS tests, yielding a specificity of 99.2%. Assay presented accuracy of 99% and a weighted kappa of 0.9, indicating an excellent performance (Table 2).

**Table 2:** Quality parameters of anti-HCV detection in dried blood spot samples using commercial EIA according to the population under analysis and their serological profile

Profile	TP	FN	TN	FP	Sensitivity% (95% CI)	Specificity% (95% CI)	PPV% (95% CI)	NPV% (95% CI)	Accuracy (95% CI)	wK (95% CI)
GI) General population (n = 1042)	6	1	1027	8	85.7 (42.1–99.6)	99.2 (98.4–99.6)	42.8 (26.0–61.4)	99.9 (99.4- 99.8)	99.1 (98.4–99.6)	0.6 (0.3- 0.8)
Tocantins/Tocantinopolis (n = 582)	3	0	575	4	100.0 (29.2–100.0)	99.3 (98.2–99.8)	42.8 (22.0–66.6)	100.0 (NA)	99.3 (98.2–99.8)	0.6 (0.2–0.9)
Rio de Janeiro/Nova Iguaçu (n = 131)	2	0	129	0	100.0 (15.8–100.0)	100.0 (97.2–100.0)	100.0 (NA)	100.0 (NA)	100.0 (97.2–100.0)	1.000 (NA)
Mato Grosso do Sul/Pantanal (n = 329)	1	1	323	4	50.0 (1.3–98.7)	98.8 (96.9–99.6)	20.0 (4.4–57.6)	99.7 (98.7–99.9)	98.5 (96.5–99.5)	0.3 (0.1–0.7)
GII) Beauty Professionals (n = 288)	3	0	284	1	100.0 (29.2–100.0)	99.6 (98.0–99.9)	75.0 (29.8–95.5)	100.0 (NA)	99.6 (98.1–99.9)	0.8 (0.6–1)
GIII) Non injecting drug users (n = 201)	1	0	197	3	100.0 (2.5–100.0)	98.5 (95.7–99.7)	25.0 (9.8–50.6)	100.0 (NA)	98.5 (95.7–99.7)	0.4 (0.1–0.9)
GIV) Ambulatory settings (n = 275)	240	4	31	0	98.3 (95.8–99.5)	100.0 (88.8–100.0)	100.0 (NA)	88.6 (74.6–95.3)	98.5 (96.3–99.6)	0.9 (0.9–1.0)
All Individuals (1806)	250	5	1539	12	98.0 (95.5–99.4)	99.2 (98.7–99.6)	95.4 (92.2–97.3)	99.7 (99.2–99.8)	99.0 (98.5–99.4)	0.9 (0.9–1.0)

FIOCRUZ Viral Hepatitis Laboratory, Biological Samples Panel, 2020

TP = True positive; FN = False-negative; TN = True negative; FP = False-positive; PPV = Positive Predictive Value; NPV = Negative Predictive Value; wK = weighted kappa index; N = number of samples; CI = confidence interval, NA = not available

In group I, 1042 individuals gave serum and DBS samples and 7 of them were anti-HCV reactive in serum and 6 were also positive in DBS, yielding a sensitivity of 85.7%. Six serum samples were tested for HCV RNA and three of them tested positive for HCV RNA (mean viral load of 2380 ± 1880.4 UI/mL). Among positive concordant samples between serum and DBS samples (n = 6), two of them had HCV RNA in their respective serum. One false negative DBS sample was found in the Pantanal sample and had HCV RNA in his/her serum. Anti-HCV was not detected in 1,035 sera and respective 1027 DBS samples giving 99.2% of specificity. The weighted Kappa was 0.6, showing substantial agreement. Low sensitivity was found among samples from Mato Grosso State, compared to samples obtained in Rio de Janeiro and Tocantins (Table 2).

In group II, 288 individuals gave sera and DBS samples, 3 were anti-HCV reactive in their sera and all of them also tested positive in DBS, with a sensitivity of 100.0%. All serum samples were tested for HCV RNA and two of them had HCV RNA detected (mean viral load of 6582 UI/mL). A sum of 285 serum samples were anti-HCV negative and 284 also tested negative in DBS demonstrating specificity of 99.6%. The weighted Kappa was 0.8, showing substantial agreement (Table 2).

In group III, 201 individuals were recruited and one of them tested positive for serum and DBS gave 100% of sensitivity. Regarding specificity, 197 individuals were negative for anti-HCV in DBS and serum samples showing 98.5% of specificity. The weighted kappa was 0.4 showing fair agreement.

In group IV, 275 individuals gave serum and DBS samples where 244 were anti-HCV reactive in serum and 240 also tested positive in DBS demonstrating a sensitivity of 98.3%. Among true positive DBS samples, 187 presented HCV RNA in serum showing mean viral load of 2.2 × 10^6^ ± 3.9 × 10^6^ UI/mL and 53 samples were HCV RNA negative in serum. Among false negative DBS samples, HCV RNA was detected in two samples (mean viral load of 5.3 × 10^5^ ± 6.8 × 10^5^ UI/mL). A total of 31 individuals were negative in serum and DBS showing 100% of specificity. Accuracy was 98.5% and the weighted kappa was 0.9 showing excellent agreement (Table 2).

### Comparison of HCV RNA detection in serum vis-à-vis DBS results

Among 255 anti-HCV reactive serum samples, 252 were submitted to PCR (97.6%) and 194 had HCV RNA in serum (77%); 191 of them also presented anti-HCV in their DBS showing a concordance of 98.4%. On the other hand, most of individuals without HCV RNA in serum presented anti-HCV in DBS (96.5%).

The following mean OD values for anti-HCV were observed in the group of HCV RNA in serum and anti-HCV in DBS (n = 191): 2.97 ± 0.17 in serum and 2.73 ± 0.59 in DBS. In the group of HCV RNA detected in serum and anti-HCV negative in DBS (n = 3), mean OD values for anti-HCV were 2.99 ± 0.009 in serum and 0.06 ± 0.04 in DBS. Samples from HCV RNA negative in serum and anti-HCV negative in DBS (n = 2) had mean OD values for anti-HCV of 1.80 ± 1.69 in serum and 0.08 ± 0.001 in DBS. Samples from HCV RNA negative in serum and anti-HCV positive in DBS (n = 56) had mean OD values of 2.70 ± 0.73 in serum and 2.14 ± 1.10 in DBS testing for anti-HCV.

### Evaluation of anti-HCV detection in the panel under analysis

Anti-HCV detection was estimated in serum and DBS for each group. Using sera, anti-HCV was detected in 0.7%, 1.0%, 0.5% and 87.3% in group I, II, III, and IV respectively. Using DBS, anti-HCV was detected in 1.3%, 1.3%, 1.9% and 87.3% in group I, II, III, and IV respectively (Additional file 1).

When anti-HCV was evaluated according to the site of recruitment in group I, anti-HCV was found to be 0.5%, 1.5%, 0.6%, using sera from Tocantins, Rio de Janeiro and Mato Grosso do Sul, respectively. Using DBS, anti-HCV was detected in 1.2%, 1.5%, 1.5%, in Tocantins, Rio de Janeiro and Mato Grosso do Sul, respectively (Additional file 2).

In samples from North and Northeast Brazil (Amazonas, Pernambuco, Ceará and Piaui States), individuals gave only DBS samples and anti-HCV prevalences were 0.4%, 0.0%, 0.0%, and 0.0%, respectively. Socio-demographic characteristics of this sample is presented in Additional file 3: Appendix.

## Discussion

This study showed the high accuracy of the laboratory procedures using DBS, with sensitivities and specificities above 98% for anti-HCV diagnosis using DBS. Similar results have been found in previous studies conducted in Brazil and abroad indicating that the method could be used for large scale anti-HCV testing [11–14, 17, 18] and confirming the feasibility of dried blood specimens for diagnosis and prevalence studies of HCV infection in context. Due to the practical infeasibility of large-scale, population-based studies comprising the comprehensive laboratory testing of several vulnerable populations living in remote areas and/or living in marginalized and underserved commumities, science will progress here by incremental gains and progressive corroboration.

When evaluating the effectiveness of anti-HCV detection in DBS according the group studied, the highest sensitivity was found in ambulatory settings (group IV) what could be the reflect of high prevalence of anti-HCV or a higher chance to be exposed to the virus among those populations. Studies conducted in high prevalence settings for anti-HCV have found a good performance of anti-HCV detection using DBS [9, 13, 16, 19].

DBS testing for anti-HCV in individuals at drug treatment centers, prison, outpatient clinics, as well as center congregating blood donors have shown sensitivities above 96% [15]. In the present study, most of DBS results from vulnerable populations/impoverished and underserved communities presented a good agreement with results from serological tests.

We also observed that anti-HCV detection in DBS was not affected by HCV RNA detection in serum, as previously observed by Mc Carron et al. [32]. This may suggest that anti-HCV could be detected in DBS samples from individuals of active HCV infection or spontaneous resolution with good sensitivity and specificity. On the other hand, Flores et al. [10] found that sensitivity of anti-HCV testing in DBS increased when HCV RNA is detected in HIV/HCV infected individuals, showing the importance of additional studies to evaluate the efficiency of anti-HCV testing in DBS before its implementation as a standard procedure.

Anti-HCV detection was identical using serum and DBS in group IV and it was similar in the groups I and II. However, high number of false positive results were observed in DBS samples from group III that is composed by NIDUs what could be the result of drug or medicines consumption without medical supervision that might interfere with anti-HCV testing. It should be recommended to confirm DBS positive results in this group by collecting serum samples to anti-HCV testing.

When anti-HCV detection was evaluated according site of recruitment in group I, same values were observed using serum or DBS in Rio de Janeiro demonstrating that DBS samples are useful tool for detecting anti-HCV. Using DBS, it was possible to report HCV prevalence of 1.5% in Brazilian Pantanal (Mato Grosso do Sul) that is the largest remaining wetland area of natural vegetation in the world. In this area, there were indigenous, riverine and quilombo communities and recently almost one-third of the area was on fire. Reis et al. [33] found 0.2% of anti-HCV prevalence among quilombo remnant communities in Midwest region of Brazil. The discrepancies in HCV prevalence and the impact of the fires that destroyed the environment reinforce the need for further epidemiological studies in this area.

Anti-HCV was tested in DBS samples from people under poverty conditions living at remote areas of North and Northeast areas (Amazonas, Pernambuco, Ceará and Piaui States). Anti-HCV detection ranges from 0 to 0.4% what is considered low compared to other studies conducted in the same area that reported prevalences from 0.9% to 6.6.% according risk behavior [2, 20, 34, 35].

Although the present study is the first in Brazil to evaluate the performance of DBS for anti-HCV testing in beauticians, NIDU, indigenous population, riverine communities, people at poverty conditions and patients referred to ambulatories at the same time, we did not investigate the impact of transport and storage conditions on DBS. However, it is possible to detect anti-HCV in samples obtaining from different regions of Brazil, most of them, far away from laboratory testing.

### Limitations

The study comprises the analysis of a panel of biological samples diverse and these populations are frequently hard-to-reach due to stigma and marginalization and/or to poverty and the difficulties of working in distant geographic locations.

As in any panel, there is here a clear trade-off: panels emulate, in a small scale, the diversity and challenges of the real world. On the other hand, they are composed by pools of convenience samples. The latter are associated with limitations secondary to biases present in any non-probability sample in terms of its generalizability and the accuracy of statistical inference [36].

Such “worlds in miniature” are key in science, but their findings must be interpreted with the necessary caution. Findings should be replicated by population-based studies for each one of the key populations. This constitute a formidable challenge from different perspectives: huge costs, complex logistics, as well as the pressing need of manpower and optimal laboratory infra-structure and availability of laboratory supplies.

## Conclusions

We concluded that DBS samples could be used for anti-HCV detection in several populations recruited in real life conditions and ambulatory settings. It has shown high sensitivity and specificity.

## Supplementary Information

**Additional** **file 1.** **Table S1.** Risk behaviors for HCV infection according to each group in the study FIOCRUZ Viral Hepatitis Laboratory, Biological Samples Panel, 2020.

**Additional** **file 2.** Table S2. Socio-demographic according to group the indigenous people from Amazon State and individuals from Northeast region. FIOCRUZ Biological Samples Panel, 2020.

**Additional** **file 3.** Appendix.

## Data Availability

The datasets generated and/or analysed during the current study are not publicly available to maintain the privacy and confidentiality of the subjects but are available from the corresponding author upon reasonable request.

## Abbreviations

CI: Confidence interval
CO: Cut-off value
EIA: Enzyme immunoassay
FN: False negative result (negative in DBS and positive in sera)
FP: False positive result (positive in DBS and negative in sera)
HBV: Hepatitis B virus
HCV: Hepatitis C virus
HIV: Human immunodeficiency virus
anti-HCV: Antibodies against hepatitis C virus
ROC: Receiver operating curve
k: Kappa index
LRNHV: National Reference Laboratory for Viral Hepatitis
NPV: Negative predictive value
n: Number of samples
OD: Optical density
PPV: Positive predictive value
SD: Standard deviation
TN: True negative result (negative in both DBS and sera)
TP: True positive result (positive in both DBS and sera)
