# Supplementary material for: Feasibility of dried blood spot for hepatitis C diagnosis in vulnerable subjects and people living in remote areas from Brazil
Source: BMC Infect Dis. 2022 Oct 27;22:804. doi: 10.1186/s12879-022-07717-4 (PMC9615222; doi:10.1186/s12879-022-07717-4)
Supplement: Supplementary file 1 — Additional file 1. Table S1. Risk behaviors for HCV infection according to each group in the study FIOCRUZ Viral Hepatitis Laboratory, Biological Samples Panel, 2020. [file 12879_2022_7717_MOESM1_ESM.docx]

S1 table. Risk behaviors for HCV infection according to each group in the study FIOCRUZ Viral Hepatitis Laboratory, Biological Samples Panel, 2020

| **Variable** | Total Population | Group I | Group II | Group III | Group IV |
| --- | --- | --- | --- | --- | --- |
|  | (n= 1,806) | (n = 1,042) | (n=288) | (n=201) | (n=275) |
|  | n (%) | n (%) | n (%) | n (%) | n (%) |
| **Previous contact** |  |  |  |  |  |
| Hepatitis or jaundice History | 346 (19.2) | 33 (3.2) | 43 (14.9) | 64 (31.8) | 206 (74.9) |
| **Factors associated to blood exposition** |  |  |  |  |  |
| History of Intravenous medicine | 186 (10.3) | - | - | - | 186 (67.6) |
| History of Surgery | 339 (18.8) | 125 (12) | 184 (63.9) | - | 30 (10.9) |
| Reporting blood transfusion | 182 (10.1) | 25 (2.4) | 17 (5.9) | 17 (8.5) | 123 (44.7) |
| Have Piercing | 131 (7.3) | 17 (1.6) | 50 (17.4) | 57 (28.4) | 7 (2.5) |
| Have tattoo | 291 (16.1) | 56 (5.4) | 89 (30.9) | 110 (54.7) | 36 (13.1) |
| Previous history of Hemodialysis | 16 (0.9) | 2 (0.2) | 1 (0.3) | 4 (2) | 9 (3.3) |
| Earring | 437 (24.2) | 126 (12.1) | 150 (52.1) | - | 161 (58.5) |
| Acupuncture | 105 (5.8) | 37 (3.6) | 24 (8.3) | - | 44 (16.0) |
| Attendance at Manicure or pedicure | 439 (24.3) | 144 (13.8) | 163 (56.6) | - | 132 (48.0) |
| Brazilian Wax | 152 (8.4) | 43 (4.1) | 74 (25.7) | - | 35 (12.7) |
| Shared blades | 265 (14.7) | 91 (8.7) | 84 (29.2) | - | 90 (32.7) |
| Shared toothbrush | 56 (3.1) | 27 (2.6) | 0 (0) | - | 29 (10.5) |
| History of invasive dental procedures | 638 (35.3) | 210 (20.2) | 170 (59.0) | - | 258 (93.8) |
| **Sexual practices** |  |  |  |  |  |
| Already had sexual intercourse | 838 (46.4) | 130 (12.5) | 258 (89.6) | 180 (89.6) | 270 (98.2) |
| Heterosexual intercourse | 890 (49.3) | 350 (33.6) | 279 (96.9) | - | 261 (94.9) |
| Homosexual intercourse | 9 (0.5) | 3 (0.3) | 2 (0.7) | - | 4 (1.5) |
| Intercourse with men and women | 9 (0.5) | 3 (0.3) | 2 (0.7) | - | 4 (1.5) |
| Sexual partners | 458 (25.4) | 100 (9.6) | 179 (62.2) | - | 179 (65.1) |
| <5 sexual partners per year | 183 (10.1) | 58 (5.6) | 60 (20.8) | - | 65 (23.6) |
| > 5 sexual partners per year | 30 (1.7) | 13 (1.2) | 11 (3.8) | - | 6 (2.2) |
| Do not or rarely use condoms in sexual intercourse | 606 (33.6) | 184 (17.7) | 164 (56.9) | 67 (33.3) | 191 (69.5) |
| Always or sometimes use condom in sexual intercourse | 344 (19.0) | 98 (9.4) | 73 (25.3) | 113 (56.2) | 60 (21.8) |
| Practice of Oral sexual intercourse | 447 (24.8) | 117 (11.2) | 197 (68.4) | - | 133 (48.4) |
| Practice of Anal sexual intercourse | 290 (16.1) | 72 (6.9) | 124 (43.1) | - | 94 (34.2) |
| History of sexually transmitted infection | 129 (7.1) | 33 (3.2) | 36 (12.5) | - | 60 (21.8) |
| Sexual partner with hepatitis or HIV | 35 (1.9) | 2 (0.2) | 7 (2.4) | 6 (3.0) | 20 (7.3) |
| **Vaccination** |  |  |  |  |  |
| Previous vaccination for HBV | 375 (20.8) | 102 (9.8) | 78 (27.1) | 42 (20.9) | 153 (55.6) |
| **Substance use** |  |  |  |  |  |
| Use of alcohol | 206 (11.4) | 146 (14.0) | - | - | 60 (21.8) |
| Use of medicines without medical supervision | 219 (12.1) | 43 (4.1) | - | - | 176 (64.0) |
| Use of illicit drugs | 273 (15.1) | 19 (1.8) | 8 (2.8) | 200 (99.5) | 46 (16.7) |
