# Supplementary material for: Feasibility of dried blood spot for hepatitis C diagnosis in vulnerable subjects and people living in remote areas from Brazil
Source: BMC Infect Dis. 2022 Oct 27;22:804. doi: 10.1186/s12879-022-07717-4 (PMC9615222; doi:10.1186/s12879-022-07717-4)
Supplement: Supplementary file 2 — Additional file 2. Table S2. Socio-demographic according to group the indigenous people from Amazon State and individuals from Northeast region. FIOCRUZ Biological Samples Panel, 2020. [file 12879_2022_7717_MOESM2_ESM.docx]

Feasibility Of Dried Blood Spot For Hepatitis C Diagnosis In Vulnerable Subjects And People Living In Remote Areas From Brazil.

Table S2. Socio-demographic according to group the indigenous people from Amazon State and individuals from Northeast region. FIOCRUZ Biological Samples Panel, 2020

| **Variables** | **Region** | | | | | | | |
| --- | --- | --- | --- | --- | --- | --- | --- | --- |
|  | **Pernambuco**  **(n= 56)** | | **Manaus (n=230)** | | **Piauí (n=83)** | | **Ceará**  **(n=53)** | |
|  | **n** | **%** | **n** | **%** | **n** | **%** | **n** | **%** |
| **Gender** |  |  |  |  |  |  |  |  |
| Female | 18 | 32.14 | 151 | 65.65 | 63 | 75.90 | 26 | 49.06 |
| Male | 7 | 12.50 | 63 | 27.39 | 20 | 24.10 | 26 | 49.06 |
| **Age (years)** | 35.1 (16.0) | | 36.2 (13.4) | | 33.2 (16.9) | | 23.0 (3.0) | |
| **Years of Education** |  |  |  |  |  |  |  |  |
| None | 0 | 0.00 | 6 | 2.61 | 16 | 19.28 | 0 | 0.00 |
| Up to 2 years | 1 | 1.79 | 32 | 13.91 | 27 | 32.53 | 0 | 0.00 |
| Up to 11 years | 1 | 1.79 | 26 | 11.30 | 13 | 15.66 | 0 | 0.00 |
| Up to 14 years | 12 | 21.43 | 47 | 20.43 | 29 | 34.94 | 46 | 86.79 |
| More than 14 years | 11 | 19.64 | 6 | 2.61 | 3 | 3.61 | 6 | 11.32 |
| **Monthly family income** |  |  |  |  |  |  |  |  |
| Low (<US$202.10) | 34 | 60.71 | 0 | 0.00 | 31 | 37.35 | 2 | 3.77 |
| Intermediate (U$202.10-606.31) | 9 | 16.07 | 0 | 0.00 | 42 | 50.60 | 9 | 16.98 |
| High(>U$606.31) | 13 | 23.21 | 0 | 0.00 | 2 | 2.41 | 42 | 79.25 |
